# Acute Myeloid Leukemia Presenting as Acute Appendicitis

**DOI:** 10.1155/2013/815365

**Published:** 2013-06-11

**Authors:** Sherri Rauenzahn, Caroline Armstrong, Brendan Curley, Sarah Sofka, Michael Craig

**Affiliations:** Department of Medicine and Section of Hematology/Oncology, One Medical Center Drive, West Virginia University, Morgantown, WV 26506, USA

## Abstract

Appendicitis in leukemic patients is uncommon but associated with increased mortality. Additionally, leukemic cell infiltration of the appendix is extremely rare. While appendectomy is the treatment of choice for these patients, diagnosis and management of leukemia have a greater impact on remission and survival. A 59-year-old Caucasian female was admitted to the surgical service with acute right lower quadrant pain, nausea, and anorexia. She was noted to have leukocytosis, anemia, and thrombocytopenia. Abdominal imaging demonstrated appendicitis with retroperitoneal and mesenteric lymphadenopathy for which she underwent laparoscopic appendectomy. Peripheral smear, bone marrow biopsy, and surgical pathology of the appendix demonstrated acute myeloid leukemia (AML) with nonsuppurative appendicitis. In the setting of AML, prior cases described the development of appendicitis with active chemotherapy. Of these cases, less than ten patients had leukemic infiltration of the appendix, leading to leukostasis and nonsuppurative appendicitis. Acute appendicitis with leukemic infiltration as the initial manifestation of AML has only been described in two other cases in the literature with an average associated morbidity of 32.6 days. The prompt management in this case of appendicitis and AML resulted in an overall survival of 185 days.

## 1. Introduction

Early and prompt diagnosis of AML has been proven to decrease morbidity and mortality [1]. Acute appendicitis has infrequently been described in the setting of known acute leukemia and is generally associated with patients receiving active chemotherapy [2]. Leukemic cell infiltration of the appendix, first reported by Rappaport in 1967, is even less-well described [3]. Despite the infrequent occurrence, appendicitis in leukemic patients is associated with a higher mortality rate [2, 4]. While appendectomy is accepted as the treatment of choice for appendicitis in patients with acute leukemia [5], diagnosis and prompt management of the leukemia have a greater impact on achieving a complete remission and, thus, overall survival. This case of acute appendicitis demonstrates the importance of maintaining a broad differential and seeking prompt diagnostic consultation.

## 2. Case Presentation

A 59-year-old Caucasian female, with no significant past medical history, presented to the surgical service for management of acute appendicitis. The patient described two days of lower abdominal pain that she noted to be worse on the right with sudden onset and increasing severity. She noted associated diarrhea two days prior to admission followed by constipation. She endorsed anorexia, nausea, and vomiting for two days. Additionally, she reported progressive shortness of breath. Patient denied chills, dysuria, or chest pain. On review of systems, patient reported an unintentional six-kilogram weight loss in the past three months, easy bruising, and night sweats. Patient stated that she had seen her primary care physician two months prior with no laboratory abnormalities reported.

**Figure 1:**
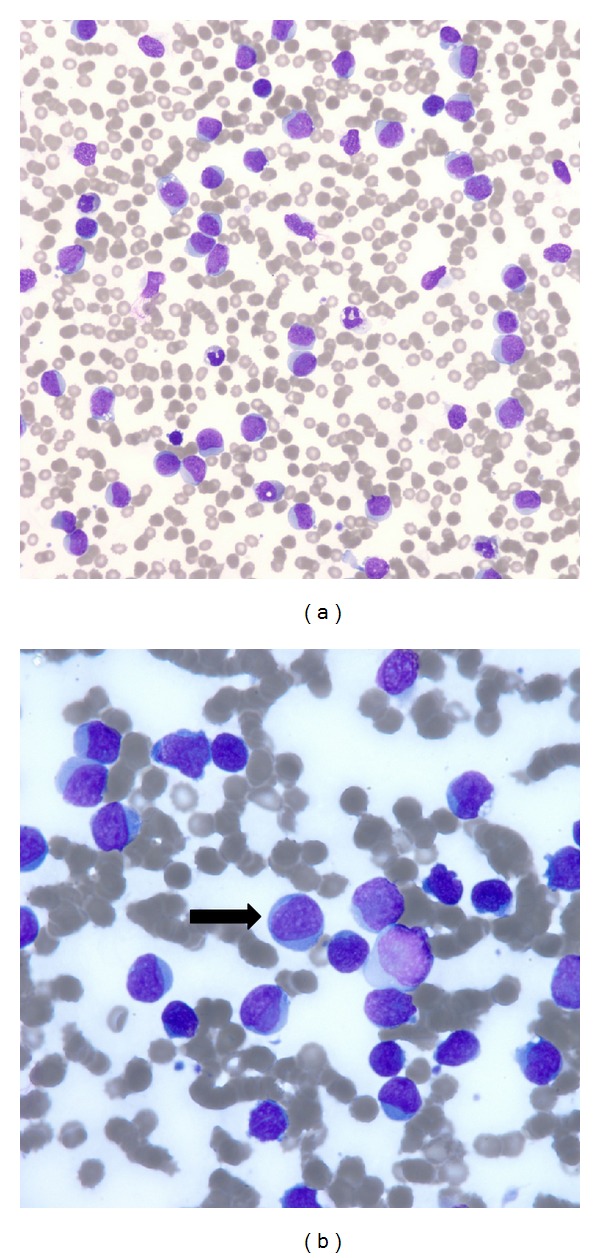
200x (a) and 500x oil (b) poweredperipheral blood smear demonstrating numerous circulating blasts (81%) without Auer rods (black arrow) consistent with acute myeloid leukemia.

On admission, the patient was found to have a white blood cell count of 159 thousand per microliter (reference range: 3.5–11.0 thousand per microliter) with 81% blasts; 11% polymorphonuclear leukocytes; 1% bands; and 7% lymphocytes and thrombocytopenia (platelet count of 76 thousand per microliter with a reference range of 140–450 thousand per microliter). Peripheral smear demonstrated numerous circulating blasts (81%) without Auer rods, anemia, and thrombocytopenia (Figure 1). Computed tomography (CT) performed at an outside facility demonstrated appendicitis with retroperitoneal and mesenteric lymph nodes. 

**Figure 2:**
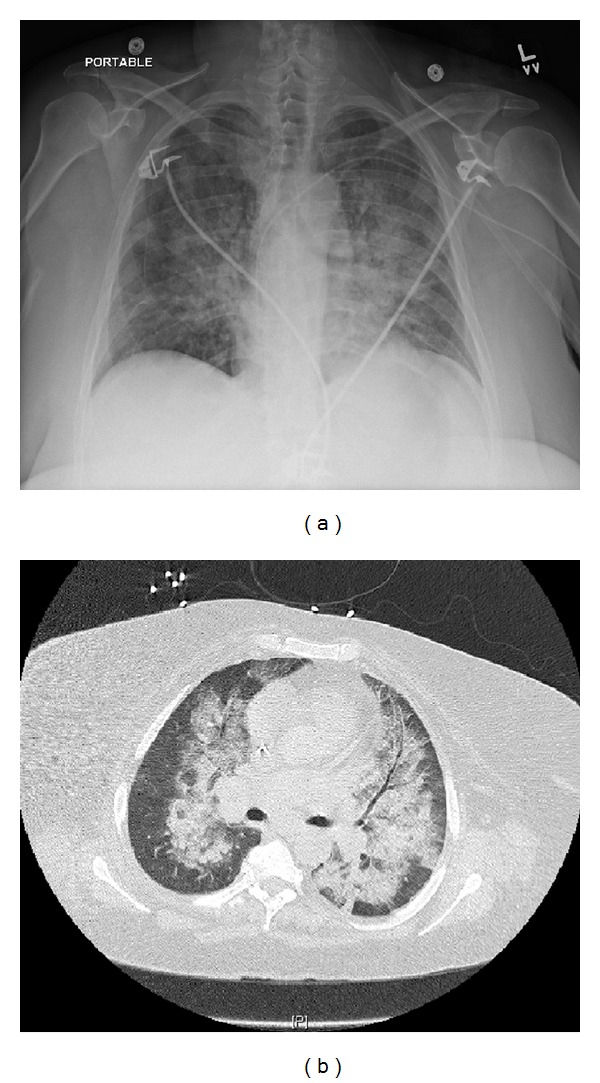
Chest X-ray (a) and computed tomography (b) demonstrating bilateral pulmonary infiltrates.

On presentation, the patient's abdominal pain was concerning for acute appendicitis and leukemoid reaction versus ischemic bowel. Given the patients anemia, leukocytosis, and thrombocytopenia, an underlying hematologic malignancy leading to hyperviscosity remained likely. Her chest X-ray and CT of the chest which demonstrated patchy bilateral ground-glass densities (Figure 2) were concerning for a bilateral pneumonia versus infiltrates secondary to congestive heart failure or leukemic infiltrates.

**Figure 3:**
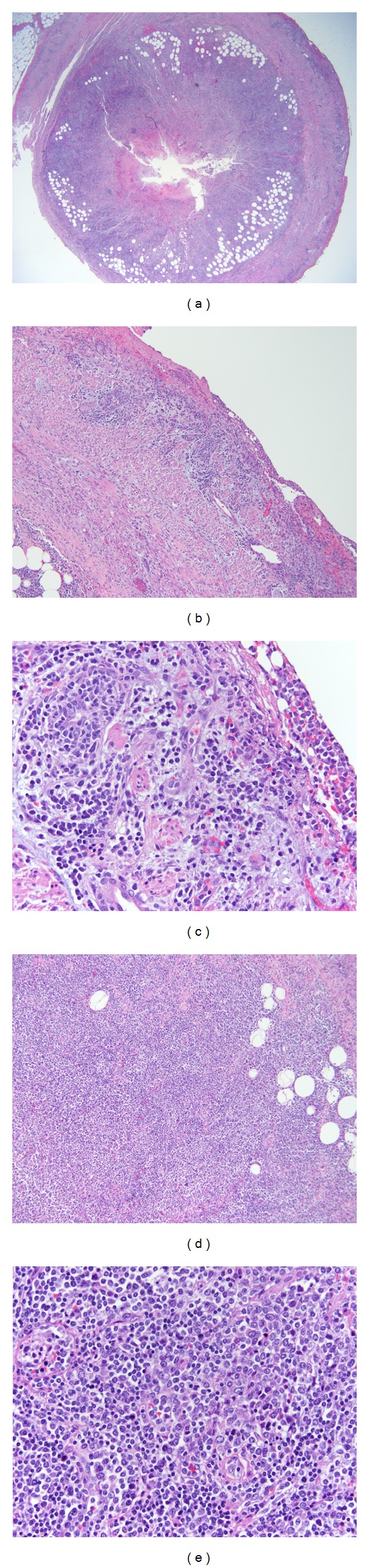
(a) 20x cross-section of the appendix specimen. (b) 100x powered and (c) 200x powered appendiceal wall demonstrating transmural blastic infiltrates. (d) 100x powered and (e) 200x powered cross-section of appendix demonstrating leukemic infiltrates.

Within hours of arrival, the patient was taken to the operating room and underwent laparoscopic appendectomy. The surgical specimen of the appendix on macroscopic review showed a tan-brown serosa with areas of hemorrhage and the lumen containing fecal material without evidence of abscess. On microscopic review the appendix revealed diffuse infiltrate of atypical larger cells consistent with blasts within the wall of the appendix (Figure 3).

**Figure 4:**
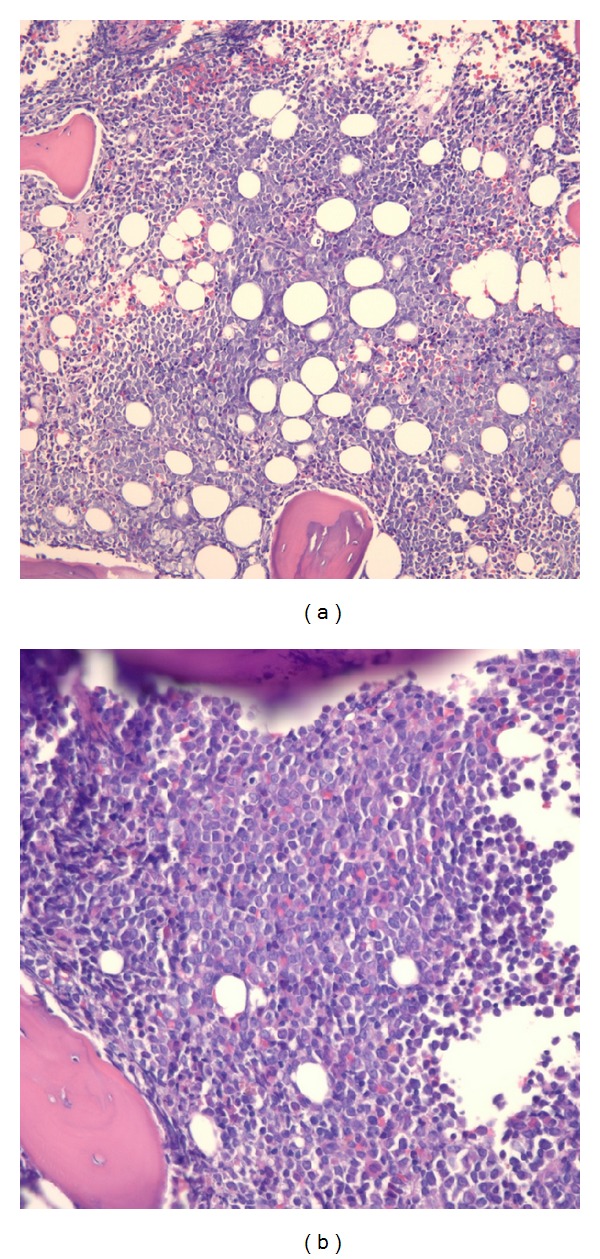
100x (a) and 200x (b) powered core bone marrow biopsy demonstrating hypercellular marrow (approximately 40–80% cellularity) with interstitial blast infiltrate (80–90% of cellularity by morphology).

Following appendectomy, she was immediately transferred to the bone marrow transplant service. She underwent unilateral bone marrow biopsy which showed FLT3-ITD- and NPM1-mutated AML with hypercellular marrow consisting of 80–90% blasts (Figure 4). Cytogenetic testing was negative for AML-associated gene rearrangements. According to WHO classification, she was found to have AML with recurrent genetic abnormalities, specifically AML with mutated NPM1.

Chemotherapy was initially delayed for one week to allow for adequate wound healing postoperatively. During the hospital course, she developed acute hypoxia. While she was initially treated with a course of antibiotics for presumed community acquired pneumonia, she failed to improve symptomatically. After several days of monitoring without response to therapy, her respiratory failure was attributed to probable leukemic infiltration of the lung; subsequently leukapheresis and hydroxyurea were initiated. Her respiratory status improved with high-dose steroids and initiation of chemotherapy with idarubicin and cytarabine (7 + 3). 

Her fourteen-day bone marrow biopsy was deferred secondary to sepsis and a decline in her performance status as demonstrated by a change in the ECOG score from 0 on admission to 3 at the time of expected repeat biopsy. She underwent a 30-day bone marrow biopsy which showed 50–60% hypercellularity and no evidence of disease recurrence. She was released from the hospital after approximately one month with her AML in remission (CR1). She returned within two months with confirmed relapsed AML on bone marrow biopsy. The patient started reinduction chemotherapy on trial comparing cytarabine/vosaroxin versus cytarabine/placebo. Following one cycle of therapy, her performance status had declined and she was no longer a candidate for additional therapy. The patient was discharged home with hospice support and expired 185 days after initial presentation.

## 3. Discussion

Leukemic infiltration has previously been documented in multiple organ systems. Wandroo et al. presented infiltrates leading to cholestatic hepatitis [6]. Leukemic infiltration of the bowel [4, 7] and pulmonary infiltration have frequently been described [1, 8, 9]. In the setting of known AML, the differential for acute abdominal pain typically includes acute appendicitis versus typhlitis. Prior case reports typically describe patients receiving chemotherapy who develop abdominal pain and are found to have suppurative appendicitis with surgical intervention [2, 5]. Of the limited cases described of acute appendicitis in patients with leukemia, less than 10 patients were noted to have nonsuppurative leukemic infiltration of the appendix proven on pathological review [2, 7, 10, 11]. The four cases described by Prolla all died within days and autopsies revealed hemorrhagic appendicitis. The average time to morbidity in the cases with demonstrated infiltrate was approximately 32.6 days. Acute appendicitis as the initial manifestation of AML, as in this case, has only been described in two other cases where the patients were found to have AML M3 and FAB M2 AML with a survival time of 30 days and 49 days, respectively [10, 11].

We report a rare case of AML presenting as acute nonsuppurative appendicitis with leukemic cell infiltration. While the associated morbidity of appendicitis and leukemia is high, this patient benefited from the prompt treatment with appendectomy and early initiation of chemotherapy. Compared to prior case reports with a mean survival of 32.6 days, our patient's length of survival was considerably longer at 185 days. This case illustrates the importance of maintaining a high suspicion for acute leukemia in the setting of a significant leukemoid reaction even if a clear acute process such as appendicitis is present.
